# Visual outcomes after endoscopic endonasal pituitary adenoma resection: a systematic review and meta-analysis

**DOI:** 10.1007/s11102-017-0815-9

**Published:** 2017-06-22

**Authors:** Ivo S. Muskens, Amir H. Zamanipoor Najafabadi, Vanessa Briceno, Nayan Lamba, Joeky T. Senders, Wouter R. van Furth, Marco J. T. Verstegen, Timothy R. S. Smith, Rania A. Mekary, Christine A. E. Eenhorst, Marike L. D. Broekman

**Affiliations:** 1 Department of Neurosurgery, Brain Center Rudolf Magnus, University Medical Center Utrecht, Heidelberglaan 100, 3584 CX Utrecht, The Netherlands; 2 Department of Neurosurgery, Cushing Neurosurgery Outcomes Center, Brigham and Women’s Hospital, Harvard Medical School, 15 Francis Street, Boston, MA 02115 USA; 3 Department of Neurosurgery, Leiden University Medical Center, Albinusdreef 2, 2333 ZA Leiden, The Netherlands; 4 MCPHS University, Boston, USA; 5 Department of Ophthalmology, University Medical Center Utrecht, Heidelberglaan 100, 3584 CX Utrecht, The Netherlands; 6 Department of Neurology, Massachusetts General Hospital, Boston, MA USA

**Keywords:** Endonasal endoscopic surgery, Pituitary adenoma, Visual outcomes, Meta-analysis

## Abstract

**Purpose:**

Patients with pituitary adenomas often present with visual deficits. While the aim of endoscopic endonasal transsphenoidal surgery (EETS) is to improve these deficits, permanent worsening is a possible outcome. The aim of this meta-analysis was to evaluate the effect of EETS for pituitary adenomas on visual outcomes.

**Methods:**

A meta-analysis was conducted according to the PRISMA guidelines. Pooled prevalence was calculated for complete recovery, improvement, and deterioration of visual field deficits, visual acuity and unspecified visual function in fixed- and random-effect models, including assessment of heterogeneity (I^2^) and publication bias (Begg’s test).

**Results:**

Out of 2636 articles, 35 case series were included in the meta-analysis. Results are described for fixed-effect models. For patients with impaired visual acuity, only one study reported complete recovery (27.2%). Pooled prevalence for improvement was 67.5% (95% CI = 59.1–75.0%), but with considerable heterogeneity (I^2^: 86.0%), and 4.50% (95% CI = 1.80–10.8%) for patients experiencing deterioration. For patients with visual field deficits, the prevalence was 40.4% (95% CI = 34.8–46.3%) for complete recovery, 80.8% (95% CI = 77.7–83.6%) for improvement, and 2.3% (95% CI = 1.1–4.7%) for deterioration. For the unspecified visual outcomes, pooled prevalence of complete recovery was 32.9% (95% CI: 28.5–37.7%), but with considerable heterogeneity (I^2^ = 84.2%). The prevalence was 80.9% (95% CI = 77.9–83.6) for improvement and 2.00% (95% CI = 1.10–3.40%) for deterioration. Random-effect models yielded similar results. Publication bias was non-significant for all the outcomes.

**Conclusion:**

While visual deficits improved after EETS in the majority of patients, complete recovery was only achieved in less than half of the patients and some patients even suffered from visual deterioration.

**Electronic supplementary material:**

The online version of this article (doi:10.1007/s11102-017-0815-9) contains supplementary material, which is available to authorized users.

## Introduction

Pituitary adenomas are the second mos t prevalent central nervous system tumors (24.6%) [1]. Patients often present with visual deficits related to chiasmal compression such as visual field deficits (46–75%) and decreased visual acuity (14–44%) [2–6]. Both of these presenting symptoms are associated with a lower health-related quality of life (HRQoL) in this patient group and are therefore seen as a clear indication for surgery [4, 7].

While the aim of surgical resection in patients presenting with visual deficits is to improve or halt further progression of these deficits, possible complications may result in permanent worsening of the same symptoms [3]. Since the introduction of endoscopic endonasal transsphenoidal surgery (EETS), postoperative visual outcomes have improved [5, 8, 9]. However, results of visual outcomes vary, and worsening of symptoms or even new deficits have been reported as well [10, 11]. As it is unclear what the effect of EETS is on visual outcomes and which determinants may influence these outcomes, variation in treatment strategy and timing of surgery between practices has been reported [4, 12]. Some centers operate on pituitary adenomas only to alleviate current visual complaints, whereas others operate with the goal to prevent the development of future symptoms and progression of existing symptoms [4, 12].

Due to variation in treatment strategy, including timing of the procedure, visual function after endoscopic endonasal surgery for pituitary tumors may vary among patients. Therefore, the primary aim of this meta-analysis was to document more precise prevalence rates of postoperative improvement, complete recovery, and deterioration of visual function. The secondary aim of this study was to identify and assess determinants, especially the effect of surgical timing, of postoperative visual functioning.

## Methods

### Search strategy

This meta-analysis was performed in accordance with the Preferred Reporting Items for Systematic Reviews and Meta-Analyses (PRISMA) statement [13]. After approval of the protocol, the following databases were searched on 07-08-2016 for relevant literature: PubMed, Embase, Cochrane, Central, CINANHL, PsycINFO, Academic Search Premier, ScienceDirect and Web of Science. The search strategy was based on the keywords: “pituitary”, “endoscopic surgery” and “vision” and search terms to exclude studies with only animals, case reports and reviews (Supplementary Table 1). In addition, articles published before 1992 were excluded, as the first report on endoscopic endonasal pituitary surgery was published in that year [14].

### Paper selection

Titles and abstracts of articles were screened by two independent authors for eligibility. Discrepancies were solved by a third author. Inclusion criteria were original peer-reviewed articles in English, describing visual outcomes after endoscopic endonasal transsphenoidal pituitary adenoma surgery in patients older than 18 years. Articles that described outcomes after pituitary apoplexy and results of resection with extended endoscopic approaches were excluded. Furthermore, case-reports, congress abstracts, commentaries and reviews were excluded. If there were overlapping cohorts, only the largest cohort was included in the review. References of selected articles were checked for possible relevant studies. Disagreements were solved by discussion.

### Data extraction

The following study characteristics were extracted from the full text articles: study design, main in- and exclusion criteria, number of participants, gender, age, tumor subtypes, tumor size, and gross total resection rate. Regarding the visual outcomes, the following data were extracted: time between diagnosis and surgery, and pre- and postoperative visual outcomes. If visual outcomes were specified by the included studies for visual acuity and visual field deficits, the number of patients that showed complete recovery, general improvement or deterioration was extracted for these specific outcomes. If outcomes were not specified for visual acuity and visual fields deficits, data were extracted for unspecified visual function. In addition, determinants of postoperative visual outcomes were extracted from the included studies.

### Study quality assessment of the included studies

Study quality was assessed using the Newcastle–Ottawa scale for cohort studies and the criteria for case series by Cowley [15, 16]. Criteria of both scales were combined and adapted for the subject of this study (Supplementary Table 2). Studies were assessed for patient selection (max. 4 points), exposure of intervention (i.e. surgery: max. 1 point) and outcome assessment (max. 5 points).

### Meta-analysis

Pooled prevalence of complete recovery, improvement and deterioration were assessed for visual acuity, visual fields deficits or unspecified visual functioning using Comprehensive meta-analysis CMA^©^ version 3. Fixed overall prevalence rates were calculated using the inverse variance method and random prevalence rates using the method of DerSimonian and Laird [17]. Fixed prevalence rates were reported in text if not further specified. Study heterogeneity was assessed by calculating I-squared values. An I-squared value >40% was deemed high. Furthermore, publication bias was assessed with Begg’s test and Egger’s test. In case of significant publication bias, a corrected fixed prevalence rate was calculated using the trim and fill method [18]. Meta-regression on covariates such as study characteristics was not possible, as these were not reported in all studies. Therefore, factors influencing visual outcomes as described in the included articles are qualitatively described. To assess the effect of study quality on the reported visual outcomes, a meta-regression was conducted with study quality as covariate.

## Results

### Study characteristics

**Fig. 1:**
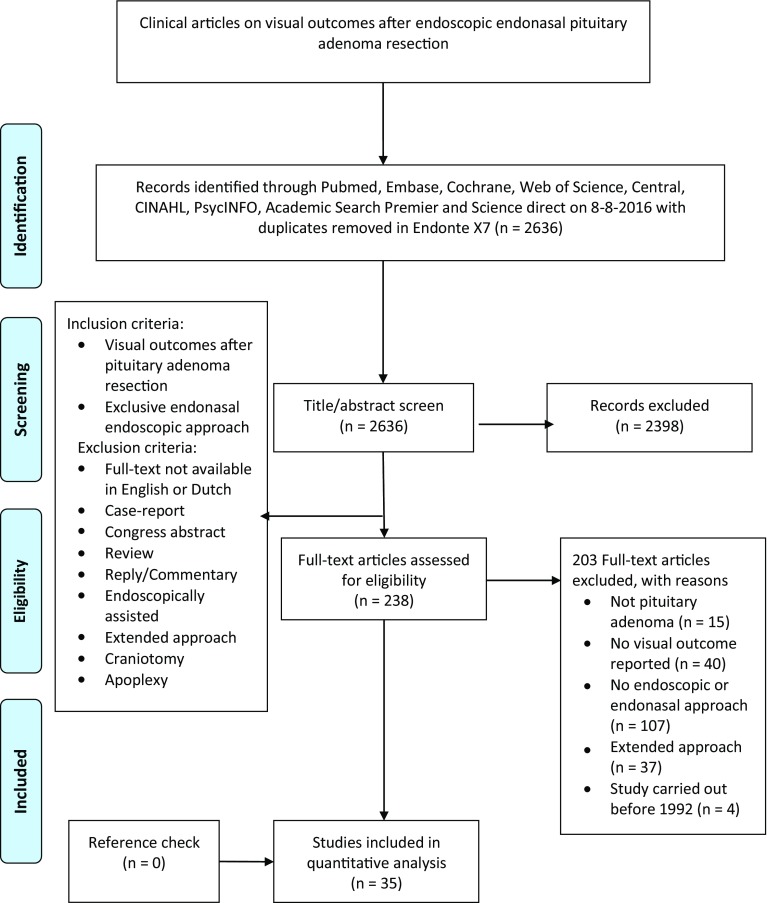
Flowchart of search strategy

After removing duplicates, 2636 articles were identified. After screening for title and abstract 2398 articles were excluded and 238 articles were reviewed full text. Afterwards, 35 studies were included in the review and meta-analysis (Fig. 1) [5, 6, 8–11, 19–47]. All studies were retrospective case series. The total number of participants ranged between 10 and 313 (median: 45). Mean age per study ranged between 35.5 and 72.5 years (median: 50) and the percentage female patients ranged between 15 and 100% (median: 45%). Seven studies included only non-functioning pituitary adenoma patients [28, 31, 33, 37, 40, 42, 46]. Gross total resection grades were reported in 26 studies and ranged between 14 and 91% (median: 63.5%) (Table 1) [5, 6, 8, 11, 20, 21, 24–26, 28–34, 36–40, 42, 43, 46, 47].

**Table 1:** Study characteristics

Author (year)	Study design	Main in- and exclusion criteria	Sample size endoscopic patients	Age in years mean (SD)/median (range)	Number female (%)	Tumor subtypes	Tumor size	Gross total resection
Studies reporting both visual acuity and visual field deficit								
Bokhari (2013)	Retrospective case series	Inclusion: PA, fully endoscopic transsphenoidal surgery (1998–2010)	79	Mean age: 56.7 (SD: 16.3)	44 (56%)	NF 39 (49%), PRL 16 (20%), GH 19 (24%), ACTH 4 (5), TSH 1 (1)	NS	50 (63%)
Campbell (2010)	Retrospective case series	Inclusion: GH producing adenomas (2005–2009)	26	Mean age: 45.7 (–)	12 (46%)	GH (100%)	84.6% ≥10 mm: 84.6%	26 (73%)
Chabot (2015)	Retrospective case series	Inclusion: adenoma size >3 cm (2009–2014)	39	Median age: 56.3 (SD: 15.6)	14 (40%)	NS	>4 cm: 15.6%	NS
Juraschka (2014)	Retrospective case series	Inclusion: PA patients (2006–2012) maximum tumor diameter in any plane ≥3 cm, and tumor volume ≥10 cm^3^. Exclusion: lack of suprasellar growth	73	Mean: 55 (SD: 15)	23 (32%)	NF 65 (89%) functioning 6 (8.2%), unknown: 2 (2.8%)	Mean: 4.09 cm	73 (24%)
Karppinen (2015)	Retrospective case series	Inclusion: NFPA patients (2000–2011) endoscopic and transnasal. Exclusion: craniectomy, re-resection, hormonally active adenomas and pituitary carcinoma	41/185	58.5 (SD: 16)	18 (44%)	NF 185 (100%)	Mean: 27 (SD: 9)	23 (41%)
Yildrim (2016)	Retrospective case series	Inclusion: NFPA pure endoscopic endonasal transsphenoidal technique (2009–2014)	160	Mean age 49 (–)	72 (45%)	NF: 160 (100%)	Mean: 2.48 cm (–)	160 (90%)
Studies only reporting visual acuity								
Constantino (2016)	Retrospective case series	Inclusion: PAs with diameter >3 cm (2010–2014)	28	Mean age: 46 (–)	11 (40%)	NS	Mean: 4.6 cm (–)	28 (14%)
De Witte (2011)	Retrospective case series	Inclusion: PA resected endoscopic transsphenoidal (2007–2010)	83	Mean age: 50 (–)	39 (47%)	NF 40 (48.3%), PRL 6 (7.2%), GH 6 (7.2%), ACTH 3 (3.6%), TSH 1 (1.2%), mixed 17 (20.5%)	73 Macroadenomas	83 (30%)
Fan (2014)	Retrospective case series	Inclusion: PA resections (2005–2010)	28	Median age: 43 (–)	17 (60%)	NF 17 (60.1), PRL 5 (17.9%), GH 3 (10.7%), ACTH 2 (7.2%) mixed: 1 (3.6%)	25 >1 cm	28 (57%)
Studies only reporting visual field deficit								
Anik (2011)	Retrospective case series	Inclusion: PA with visual field deficits. Exclusion: comorbidity that can influence vision (2009–2010)	72	Mean age: 45.7 (SD: 10.9)	43 (57%)	NF 42 (58.3), PRL 6 (8.3%), GH 22 (30.5%), ACTH 2 (2.8%)	NS	NS
Akin (2016)	Retrospective case series	Inclusion: prolactinomas reected endonasally (2006–2012)	142	Mean age: 35.5 (SD: 13.3)	76 (54%)	PRL 142 (100%)	113 Macroadenomas, 10 giant adenomas	NS
Cappabianca (1999)	Retrospective case series	Inclusion: pituitary adenoma, microscopic (1996) endoscopic (1997)	10/30	Range: 33–67	4 (40%)	NF 5 (50%), GH 5 (50%)	7 Microadenomas	9 (90%)
Chi (2013)	Retrospective case series	Inclusion: PA with endonasal extension (2011–2012)	80	Mean age: 51 (–)	35 (44%)	NF 24 (30%), PRL 26 (32.5%), GH 9 (11.3%), ACTH 3 (3.8%), TSH 3 (3.8%), mixed 5 (6.3%)	16 Microadenomas	80 (64%)
Cho (2002)	Retrospective case series	Inclusion: cohort of endoscopic and sublabial resection of prolactinomas (1996–2000)	22/44	Mean age: 45 (–)	22 (100%)	PRL 22 (100%)	NS	NS
Dallapiazza (2015)	Retrospective case series	Inclusion: NFPA patients with >5 year follow-up	80	Mean age: 57 (SD: 13)	42 (53%)	NF 80 (100%)	31 > 3.0 cm	80 (71%)
Dehdashti (2008)	Retrospective case series	Inclusion: PA patients, purely endoscopic endonasal operation (2004–2007). Exlcusion: very large pituitary adenomas and extended approaches	200	Mean age 50 (–)	109 (55%)	NF 111 (55.5%), PRL 25 (12.5%), GH 34 (17%), ACTH 27 (13.5), TSH 3 (1.5)	NS	182 (97%)
D’Haens (2009)	Retrospective case series	Inclusion: pituitary adenoma resected microscopic (1995–2001), endoscopic (2001–2007)	60/120	Mean age 37 (–)	41 (68%)	PRL 29 (48%), GH 13 (23%), ACTH 16 (27%), TSH 2 (3%)	NS	NS
Leach (2010)	Retrospective case series	Inclusion: sellar lesions (2005–2007) operated endoscopically	89/125	NS	NS	NF 67 (54%), PRL 9 (7%), GH 22 (18%), ACTH 10 (8%), craniopharyngioma 4 (3%), Other 7 (6%)	NS	NS
Minet (2008)	Retrospective case series	Inclusion: PA with >6 month follow-up (2003–2005)	31/71	Mean age: 51.4 (17.2) (endonasal)	14 (45%) (endonasal)	NF 27 (87%) PRL 2 (7%), TSH: 1 3 (%), GH: 1 (3%)	NS	NS
Nakao (2011)	Retrospective case series	Inclusion: giant NFPA endonasal resection (2000–2008)	43	Mean age: 55 (–)	20 (47%)	NF 43	Mean diameter: 47.8 mm (SD 1.2)	20 (47%)
Paluzzi (2014)	Retrospective case series	Inclusion: purely endoscopic operated PA patients (2002–2011)	555	NS		NF 360 (69.2), PRL 53 (10%), GH 49 (10%), ACTH 58 (11%)	89.4% >1 cm	359 (65%)
Sheehan (1999)	Retrospective case series	Inclusion: NFPA endonasal resection (1995–1997)	26/70	Mean age: 59.2 (SD: 15.1)	8 (31%)	NS	Volume: 11.0 (SD: 6.9) cm^3^	NS
Studies reporting unspecified visual function								
Chohan (2016)	Retrospective case series	Inclusion: PAs with size >10 cm^3^ or cross sectional length >4 cm (2003–2014)	62	Mean age 54 (–)	27 (44%)	NS	Median volume: 13.74 cm^3^	62 (47%)
Cusimano (2012)	Retrospective case series	Inclusion: giant pituitary adenomas (>10 cm^3^) resected endoscopically or by craniotomy or microscopically (1994–2001)	29/72	Mean age 50 (SD: 15)	13 (45%)	NF 25 (83%), functioning 4 (17%)	Mean: 4.0 cm	26 (91%)
Gondim (2014)	Retrospective case series	Inclusion: giant pituitary adenomas >4 cm (1998–2011)	50	Mean age: 48.2 (–)	17 (34%)	NF 42 (80%) GH 5 (10%) PRL 3 (6%)	>4 cm	50 (38%)
Gondim (2015)	Retrospective case series	Inclusion: NFPA exclusion: previous surgery, age >70 (2000–2012)	55	Mean age: 72.5 (SD: 2)	23 (42%)	NF 55 (100%)	Mean size 33 mm (SD: 23)	55 (78%)
Ferreli (2014)	Retrospective case series	Inclusion: NFPA with cavernous sinus invasion. Exclusion: grade 1 and 2 according to Knosp; patients who had previously been treated with radiotherapy in the pituitary region; patients with follow-up shorter than 36 months. (2000–2010)	56	median: 59 (37–79)	20 (36%)	NF 56 (100%)	34 > 1 cm, 22 > 4 cm	17/56 (30%)
Han (2013)	Retrospective case series	Inclusion: PA, endoscopic endonasal approach (2009–2012)	250	Mean age: 43.8 (–)	151 (60%)	NF 147 (58.8%), PRL 33 (13.2%), GH 42 (16.8%), ACTH 20 (8%), TSH 3 (1.2%), mixed 5 (2%)	116 Macroadenomas	250 (86%)
Jho (1997)	Retrospective case series	Inclusion: PA endoscopic endonasal approach (1993–1995)	15	Median age: 43 (range 17–88)	9 (60%)	NF 6 (40%), PRL 5 (33.3%), ACTH 3 (20%), adenoma 1 (6.7%)	NS	NS
Koutourousiou (2013)	Retrospective case series	Inclusion: giant PA with diameter >4 cm (2002–2011)	54	Mean age: 53 (–)	8 (15%)	NS	Mean: 32.88 cm^3^ (–)	11 (20%)
Kuo (2016)	Retrospective case series	Inclusion: giant PA size (> 4 cm in at least 1 direction or estimated tumor volume > 10 cm^3^) (2002–2009)	38	Mean age: 51 (SD: 13)	23 (60%)	NS	Mean: 3.2 cm^3^ (SD: 4.6)	38 (21%)
Marenco (2011)	Retrospective case series	Inclusion: PA: >65 years old, non-functioning (2001–2013)	25	Mean age: 72 (SD: 5)	14 (56%)	NF 25 (100%)	Mean: 3.4 cm (SD: 7.3)	25 (31%)
Sabry (2015)	Retrospective case series	Inclusion: endonasally operated PA	40	Median: 48 years (range 18–81)	18 (45%)	NS	Volume: 9.48 cm^3^ (SD 12.7)	33 (83%)
Wongsirisuwan (2014)	Retrospective case series	Inclusion: PA operated with keyhole and endonasal approach (2003–2013)	38/130	NS	NS	NS	NS	NS
Zhan (2015)	Retrospective case series	Inclusion: PA patients resected endoscopically (2008–2014)	313	NS	125 (39%)	NS	NS	239 (75%)

*NS* not specified, *SD* standard deviation, *NF* non-functioning, *ACHT* adrenocorticotrophic hormone, *GH* growth hormone, *TSH* thyroid stimulating hormone, *PRL* prolactinoma, *PA* pituitary adenoma

### Visual outcomes

Results of postoperative visual outcomes are reported in Supplementary Table 2 for each study. Pooled prevalence rates for visual acuity, visual fields deficits, and unspecified visual functioning are reported in Table 2.

**Table 2:** Outcomes of the meta-analysis

	Fixed model prevalence rate (%)	95% CI (%)	Random model prevalence rate (%)	95% CI (%)	I-squared value (%)	p value heterogeneity	Egger’s test (p value)	Begg’s test (p value)
Visual acuity								
Improvement	67.5	59.1–75.0	77.2	54.4–90.6	86.0	<0.01	0.22	0.14
Deterioration	4.5	1.8–10.8	4.5	1.8–10.8	0.00	0.62	1.00	0.49
Complete restoration^a^								
Visual field deficit								
Improvement	80.8	77.7–83.6	83.0	77.1–87.7	62.3	<0.01	0.25	0.14
Deterioration	2.3	1.1–4.7	2.3	1.1–4.7	0.00	0.93	0.21	0.16
Complete restoration	40.4	34.8–46.3	37.8	26.4–50.8	0.00	73.2	0.72	0.40
Unspecified vision								
Improvement	80.9	77.9–83.6	81.7	77.1–85.6	38.8	0.08	0.10	0.15
Deterioration	2.0	1.1–3.4	2.0	1.1–3.4	0.00	0.96	1.00	0.74
Complete restoration	32.9	18.5–37.7	39.6	23.2–58.6	84.2	<0.01	0.50	0.53

^a^Complete restoration of visual acuity was only reported in one study and therefore a meta-analysis was not possible

### Visual acuity

Patients presented with visual acuity complaints in 14–84% of cases (Supplementary Table 3) [5, 36]. Ten studies evaluated postoperative visual acuity in their case series [5, 6, 9, 20, 22, 25, 30, 36, 37, 46]. Pooled prevalence of overall improvement was 67.5% (95% CI: 59.1–75.0%, I^2^: 86.0%, n = 163/219). Pooled prevalence of deterioration was 4.5% (95% CI: 1.8–10.8%, I^2^: 0.00%, n = 3/122) (Table 2). One study reported complete recovery of visual acuity in 9 out of 33 patients [46]. No significant publication bias was identified. One study described a significant improvement in mean Snellen test score for both the right eye [preoperative: 0.72 (SD: 0.14); postoperative 0.83 (SD: 0.16); p < 0.01] and left eye [preoperative: 0.76 (SD: 0.16); postoperative 0.85 (SD: 0.14); p = 0.04] [19].

### Visual field deficit

Patients presented with visual field deficits in 28–100% of cases (Supplementary Table 3) [5, 41, 42]. Nineteen studies described outcomes of visual field deficits [5, 8–11, 19–23, 27–29, 36, 37, 41, 42, 44, 46]. The overall prevalence was 40.4% (95% CI: 34.8–46.3%, I^2^: 0.00%, n = 122/346) for complete recovery, 80.8% (95% CI: 77.7–83.6%, I^2^: 62.3%, n = 678/817) for overall improvement, and 2.3% (95% CI: 1.1–4.7%, I^2^: 0.00%, n = 3/398) for deterioration in which patients showed a decreased visual functioning postoperatively (Table 2). One study described a mean Goldmann Humphrey VFD score of 1.1 (SD: 1.1) preoperatively, compared to 0.2 (SD: 0.5) postoperatively, indicating a clear improvement (p < 0.05) [41]. No significant publication bias was identified.

### Unspecified vision

In studies that did not specify type of visual problems, 17–100% of the patients presented with visual problems (Supplementary Table 3) [31, 39]. Unspecified outcomes of visual improvement were reported in 13 studies [24, 26, 31–35, 38–40, 43, 45, 47]. Pooled prevalence for complete recovery was 32.9% (95% CI: 28.5–37.7%, I^2^: 84.2%, n = 139/416). Improvement was reported in 80.9% (95% CI: 77.9–83.6%, I^2^: 38.8%, n = 648/788). Deterioration occurred in 2.0% (95% CI: 1.1–3.4%, I^2^: 0.00%, n = 10/721). No significant publication bias was identified.

### Study-quality assessment

The majority of studies (83.3%) had a high risk of bias due to suboptimal methodology or poor reporting (Table 3). While all studies clearly described the surgical approach (exposure of intervention), only one study scored all points for selection and description of included patients and only one study for the description, interpretation, and discussion of the outcomes (outcome assessment) [31, 40]. However, study quality was associated with visual outcomes in a meta-regression (all p < 0.05).

**Table 3:** Study quality assessment

Author (year)	Selection (max. 4 points)	Exposure (max. 1 points)	Outcome (max. 5 points)	Total points (max. 10)
Visual acuity and visual field studies				
Bokhari (2013)	2	1	3	6
Campbell (2010)	2	1	3	6
Chabot (2015)	3	1	1	7
Juraschka (2014)	3	1	2	6
Karppinen (2015)	3	1	4	8
Yildrim (2016)	3	1	2	6
Visual acuity studies				
Constantino (2016) [25]	3	1	1	5
De Witte (2011) [6]	2	1	2	5
Fan (2014) [30]	1	1	1	3
Visual field studies				
Anik (2011) [19]	3	1	4	8
Akin (2016) [10]	3	1	2	6
Cappabianca (1999) [21]	2	1	1	4
Chi (2013) [8]	1	1	2	4
Cho (2002) [23]	3	1	1	5
Dallapiazza (2015) [28]	3	1	1	5
Dehdashti (2008) [29]	2	1	2	5
D’Haens (2009) [27]	2	1	2	5
Leach (2010) [9]	1	1	3	5
Minet (2008) [41]	2	1	3	6
Nakao (2011) [42]	2	1	3	6
Paluzzi (2014) [11]	2	1	4	7
Sheehan (1999) [44]	2	1	3	6
Visual function, unspecified				
Chohan (2016) [24]	3	1	1	5
Cusimano (2012) [26]	3	1	1	5
Gondim (2014) [32]	2	1	5	7
Gondim (2015) [33]	2	1	1	4
Ferreli (2014) [31]	3	1	1	5
Han (2013) [34]	3	1	2	6
Jho (1997) [35]	3	1	2	5
Koutourousiou (2013) [38]	2	1	2	5
Kuo (2016) [39]	3	1	2	6
Marenco (2011) [40]	4	1	3	8
Sabry (2015) [43]	2	1	3	6
Wongsirisuwan (2014) [45]	3	1	1	5
Zhan (2015) [47]	1	1	2	4
Median scores	2 points	1 point	2 points	6 points
Studies scoring maximum points	2.8%	100%	2.8%	

### Factors influencing visual outcomes

Factors affecting postoperative visual functioning may be procedure, symptom, tumor or patient related. Regarding procedural circumstances, increasing surgeon experience was positively associated with postoperative visual field deficit improvement in three studies (early groups: 75–86%; late groups: 90–100%) [5, 8, 9]. Furthermore, extend of resection was associated with poor visual field deficit outcomes in one study (p = 0.01), while three studies found no significant relation, both for visual acuity and visual field deficit outcomes [22, 36, 40, 42].

Regarding symptoms, longer duration of visual field deficits led to worse visual outcomes in two studies, while one study did not find a relation [19, 42]. In the first study, patients with complete recovery had a shorter visual field deficit symptoms (14.7 weeks, SD: 10.5), than patients with partial recovery (50.1 weeks, SD: 29.1) and patients with no recovery (92.4 weeks, SD: 15.4) (p < 0.01) [19]. Also, one study demonstrated that severity of visual field deficit symptoms was associated with worse visual outcomes in a multivariable analysis [48]. Finally, patients with bilateral visual field deficit had significantly better outcomes (p = 0.025) [22].

Regarding tumor related factors, functioning (growth hormone producing tumors: 71%, prolactinomas 63–75%) and non-functioning pituitary tumors (43–100%) seem to have similar visual outcomes with regard to visual field deficits and unspecified visual improvement [8, 10, 19, 28, 31, 32, 37, 42, 44, 46]. One study found a significant relation between suprasellar extension and worse visual outcomes based on patient reported outcome measures, where another does not find a relation with visual field deficits [42, 49]. Also, tumor size does not seem to have a great influence on both postoperative visual acuity and visual field deficits as seen in two studies with larger tumors (>3 cm in diameter and a volume >10 cm^3^) where improvement was seen in 69 and 70% of cases [22, 24].

With regard to patient characteristics, the influence of age on visual outcomes shows conflicting results in four studies [47]. One group reported no significant differences between patients younger and older than 65 for unspecified visual symptoms [33, 40, 47, 48]. Similarly, two studies reported outcomes of patients older than 65 and 70 years with unspecified visual improvement in 71 and 87% of cases, respectively [33, 40]. However, one study associated younger age with visual field deficit improvement in a multivariate analysis [48].

## Discussion

This meta-analysis showed that pituitary adenoma resection in patients with preoperative visual symptoms considerably improves these symptoms in the majority of cases. Furthermore, up to 30% of patients have complete recovery of their vision. However, deterioration is not uncommon either, occurring in up to 4% of cases. While the considerable improvement of visual deficits is a clear indication for EETS in patients with pituitary adenomas, a better understanding of the factors that influence these outcomes may result in even better postoperative results. These factors can be procedure, symptom, tumor, and patient related.

Factors related to the procedure may influence visual outcomes in several ways, such as experience with EETS. Although visual outcomes may improve with increased experience, this may be balanced out by selection of more complex cases. Despite this, surgeon experience was found to be a significant influence on visual outcomes in three studies [5, 8, 9]. However, gross total resection was not associated with improved visual outcomes, probably because also partial resection will result in decompression of the optic nerve/chiasm [22, 36, 40, 42]. Besides surgeon experience, surgeon preference for preventive surgery or surgery after development of visual deficits may also result in different outcomes.

Apart from procedure related factors, the duration and severity of preoperative visual symptoms may also affect postoperative outcomes [19, 48, 50]. For instance, one study found that patients with long lasting (≥1 year) preoperative visual symptoms showed significantly worse visual outcomes after microscopic resection [50]. As the timing of the postoperative visual examination varied greatly among the studies and visual symptoms may improve after longer periods of time, this may also be of influence [9–11, 22, 31, 34, 37, 43, 51, 52].

Several patient and tumor characteristics may also affect visual outcomes. Increasing age does not seem to be associated with worse outcomes [33, 40, 47, 48]. Tumor size and whether the tumor is hormone producing does not seem to greatly alter the visual outcomes [10, 20, 22–25, 36, 38, 39]. The lack of influence from tumor size on visual outcomes may possibly be explained by the slow growth of these tumors, which gives the optic nerve/chiasm time to adapt. However, indication and aim of surgery for functioning and non-functioning pituitary adenomas differs principally, as surgical indication for functioning pituitary adenomas is often not visual deficits. Therefore, different visual outcomes of surgery may be expected when comparing these two groups.

One other meta-analysis for pituitary adenomas by DeKlotz et al. reported an overall visual improvement of 71% (95% CI: 59–83%) in patients operated endoscopically based on nine studies, which is similar to our findings. This was also significantly higher than the patients operated microscopically (56%, 95% CI: 40–72%) [53].

Several other tumors in the sellar region may cause impaired vision. Patients with meningiomas showed improvement of vision after endoscopic resection in 87% of cases in one meta-analysis [54]. Another review evaluating anterior skull base meningiomas reported improvement in 69.1% of cases, but deterioration in 12.7% of cases, the latter being considerably higher than our findings [55]. A meta-analysis of visual outcomes after endoscopic surgery for craniopharyngioma showed improvement in 56.2% and deterioration 1.7% of cases [56]. Similarly, another meta-analysis for craniopharyngiomas even found an improvement in 85.5% of cases and deterioration in 2.3% [57]. With regard to this variation in results, it is not entirely unlikely that different tumors with different characteristics offer different visual outcomes after endoscopic resection. As a result, while meningioma resection seems to be associated with superior visual outcomes compared pituitary adenoma resection, craniopharyngioma resection seems to offer slightly inferior outcomes [54, 56, 57].

While there are over 30 existing questionnaires measuring patient-reported visual function and vision-related HRQoL, no studies were identified measuring these outcomes in endoscopically operated pituitary patients [58]. Measurement of patient-reported visual function and HRQoL is of added value as it is known that physician-reported outcomes and patient-reported outcomes may poorly correlate and because these questionnaires measure not only visual deficits but also consequences of these deficits on daily life [59].

The main limitation of this meta-analysis is the inability to examine reported patient characteristics as a possible source of heterogeneity, because not all pituitary adenoma patients present with visual problems. Patient characteristics were reported for the whole population, not specifically for patient presenting with visual symptoms. Thus, no clear factors were identified that could contribute to better outcomes. Furthermore, as the studies identified only described visual recovery with relatively short follow-up, long-term visual outcomes could not be studied.

Even though our study shows that EETS for pituitary adenoma improves visual complaints for most patients, incomplete recovery or even worsening of symptoms is not uncommon. Therefore, future research should be focused on identifying risk factors for incomplete recovery or even deterioration of vision with adequate follow-up. The role of surgical experience, patient characteristics, tumor characteristics, and severity and duration of visual symptoms should be studied (preferably prospectively) to identify the optimal timing of, and indications for, EETS for pituitary adenomas.

## Conclusion

ETTS for pituitary adenomas improves visual deficits considerably in the majority of cases in the postoperative period. However, EETS only results in complete recovery in 30–40% of cases and 4% of patients even experience deterioration of visual symptoms. This is particularly relevant, as visual symptoms often form the indication of surgery. Future research should therefore focus on identifying risk factors for incomplete recovery and deterioration of vision in order to improve visual outcomes.

## Electronic supplementary material

Below is the link to the electronic supplementary material.

Supplementary material 1 (DOCX 126 KB)

Supplementary material 2 (DOCX 77 KB)

Supplementary material 3 (DOCX 110 KB)
