# Supplementary material for: Visual outcomes after endoscopic endonasal pituitary adenoma resection: a systematic review and meta-analysis
Source: Pituitary. 2017 Jun 22;20(5):539–52. doi: 10.1007/s11102-017-0815-9 (PMC5606952; doi:10.1007/s11102-017-0815-9)
Supplement: Supplementary file 1 — Supplementary material 1 (DOCX 126 KB) [file 11102_2017_815_MOESM1_ESM.docx]

**Supplementary Table 1: Search Strategy**

| **Search terms:** pituitary, surgery, visual outcomes and terms to exclude studies with only animals, reviews and case reports |
| --- |
| **Search string:**  (("Pituitary Neoplasms"[Mesh] OR "Pituitary Neoplasm"[tw] OR "Pituitary Neoplasms"[tw] OR "Pituitary Tumors"[tw] OR "Pituitary Tumor"[tw] OR "Pituitary Tumours"[tw] OR "Pituitary Tumour"[tw] OR "Pituitary Adenoma"[tw] OR "Pituitary Adenomas"[tw] OR "Pituitary Carcinoma"[tw] OR "Pituitary Carcinomas"[tw] OR "Cancer of the Pituitary"[tw] OR "Pituitary Cancer"[tw] OR "Pituitary Cancers"[tw] OR "Nelson Syndrome"[tw] OR "Prolactinoma"[tw] OR "Prolactinomas"[tw] OR prolactinoma*[tw] OR "Hypophysis Adenoma"[tw] OR "Hypophysis Adenomas"[tw] OR "Hypophyseal Neoplasms"[tw] OR "Hypophyseal Tumors"[tw] OR "Hypophyseal Tumor"[tw] OR "Hypophyseal Tumours"[tw] OR "Hypophyseal Tumour"[tw] OR "Hypophyseal Adenoma"[tw] OR "Hypophyseal Adenomas"[tw] OR "Hypophyseal Carcinoma"[tw] OR "Neurohypophyseal Tumors"[tw] OR "Neurohypophyseal Tumor"[tw] OR "Adenohypophyseal Neoplasm"[tw] OR "Adenohypophyseal Tumors"[tw] OR "Adenohypophyseal Adenomas"[tw] OR "Adenohypophyseal Carcinoma"[tw] OR (Pituitar*[ti] AND ("Neoplasms"[ti] OR "Neoplasm"[ti] OR "Neoplasms"[ti] OR "Tumors"[ti] OR "Tumor"[ti] OR "Tumours"[ti] OR "Tumour"[ti] OR "Adenoma"[ti] OR "Adenomas"[ti] OR "Carcinoma"[ti] OR "Carcinomas"[ti] OR "Cancer of the Pituitary"[ti] OR "Cancer"[ti] OR "Cancers"[ti]))) **AND** (endoscop*[tw] OR "Endoscopy"[mesh:noexp] OR "Neuroendoscopy"[mesh] OR neuroendoscop*[tw] OR adenectom*[tw] OR transsphenoid*[tw] OR "Sphenoid Bone/surgery"[mesh] OR Endonasal*[tw] OR Endo-nasal*[tw] OR "Transanal Endoscopic Surgery"[mesh] OR Transanal Endoscop*[tw] OR Surg*[tw] OR resect*[tw] OR operation*[tw] OR operativ*[tw] OR "Surgical Procedures, Operative"[mesh] OR "Neurosurgical Procedures"[Mesh:NoExp] OR "Neurosurgery"[mesh] OR "Brain/surgery"[Mesh] OR neurosurg*[tw] OR surgery[subheading]) **AND** (Visual*[tw] OR vision*[tw] OR ("visual"[tw] AND "field"[tw] AND defect*[tw]) OR hemianopsi*[tw] OR hemianopia*[tw] OR "Hemianopsia"[mesh] OR (("altitudinal"[tw] OR "localized"[tw]) AND defect*[tw]) OR scotoma*[tw] OR "Scotoma"[mesh] OR "Hemifield Test"[tw] OR "Hemifield Tests"[tw] OR "Vision, Ocular"[mesh] OR "Vision, Low"[mesh] OR "Visual Fields"[mesh] OR "Visual Perception"[mesh] OR "Visual Acuity"[mesh] OR blind*[tw] OR "Blindness"[mesh] OR "eyesight"[tw] OR "afferent pupillary defect"[tw] OR "Visual Field Tests"[mesh] OR (("eye"[tw] OR "ocular"[tw]) AND manifestation*[tw]) OR "Eye Manifestations"[mesh] OR "perimetry"[tw] OR "Humphrey Visual Field"[tw] OR ("Frequency"[tw] AND "Dousing"[tw] AND "Technology"[tw]) OR "Vision Disorders"[Mesh] OR "Alice in Wonderland Syndrome"[tw] OR "Amblyopia"[tw] OR "Amaurosis Fugax"[tw] OR "Deaf-Blind"[tw] OR "Color Vision Defects"[tw] OR "Color Vision Defect"[tw] OR "Diplopia"[tw] OR "Photophobia"[tw])) **AND** (english[la] OR dutch[la]) NOT ("Animals"[mesh] NOT "Humans"[mesh]) NOT ("Case Reports"[Publication Type] NOT ("Clinical Study"[ptyp] OR "review"[ptyp] OR review*[ti])) |
| **The search strategy was adapted for the following electronical databases: Embase, Web of Science, CINAHL, PsychInfo, Academic Search Premier, COCHRANE and ScienceDirect.** |
