# Supplementary material for: Visual outcomes after endoscopic endonasal pituitary adenoma resection: a systematic review and meta-analysis
Source: Pituitary. 2017 Jun 22;20(5):539–52. doi: 10.1007/s11102-017-0815-9 (PMC5606952; doi:10.1007/s11102-017-0815-9)
Supplement: Supplementary file 2 — Supplementary material 2 (DOCX 77 KB) [file 11102_2017_815_MOESM2_ESM.docx]

Supplementary Table 2: Study quality assessment

| **Criteria** | | | **Description** |
| --- | --- | --- | --- |
| **Selection (max. 4 points)** | **Description and representativeness of the exposed subjects** | **Consecutive inclusion (1 point)** | A consecutive series of patients is included in the study. |
|  |  | **Clear description in- and exclusion criteria (1 point)** | A clear description is given of the in- and exclusion criteria, including the diagnosis (e.g. histological, imaging etc.). |
|  |  | **Patient and tumor characteristics appropriately described (1 point)** | Description of patient- and tumor characteristics is given: age, gender, previous operations and tumor type. |
|  |  | **Visual comorbidities described (1 point)** | Visual comorbidities at time of surgery are described. |
| **Exposure (max. 1 points)** | **Intervention** | **Clearly described surgical approach  (1 point)** | The surgical approach is clearly described or references to articles describing the surgical approach are given. |
| **Outcome (max. 5 points)** | **Outcome assessment** | **Measurement criteria described (1 point)** | A clear description of the used techniques to assess visual outcomes or references to articles describing the technique is given. |
|  |  | **Clinically relevant outcomes described  (1 point)** | Visual outcomes are described as clinically relevant (i.e. minimal important change or interpretation of authors). |
|  |  | **Pre- and postoperative outcome assessment (1 point)** | Visual functioning measured both before and after surgery. |
|  |  | **Timing of outcome described (1 point)** | Description of timing of visual outcome assessment is given. |
|  |  | **Percentage < 10% (1 point)** | Less than 10% of patients are lost to follow-up. |
