# Supplementary material for: Visual outcomes after endoscopic endonasal pituitary adenoma resection: a systematic review and meta-analysis
Source: Pituitary. 2017 Jun 22;20(5):539–52. doi: 10.1007/s11102-017-0815-9 (PMC5606952; doi:10.1007/s11102-017-0815-9)
Supplement: Supplementary file 3 — Supplementary material 3 (DOCX 110 KB) [file 11102_2017_815_MOESM3_ESM.docx]

Supplementary Table 3: Visual Outcomes

| **Author (year)** | **Time between diagnosis and operation** | **Moment of measurement postoperative** | **Preoperative** | **Postoperative** | | | |
| --- | --- | --- | --- | --- | --- | --- | --- |
|  |  |  |  | Improvement | Normalization | Deterioration | Stable |
| **Studies reporting visual acuity (VA) and visual field (VF)** | | | | | | | |
| Bokhari (2013)^5^ | NS | NS | VF: 22 (28%) VA: 11 (14%) | VF: 17 (77%)  VA: 6 (55%) | VF: 4 (15%)  VA: - | VF: 1 (5%)  VA: - | VF: - VA: 5 (45%) |
| Campbell (2010)^20^ | NS | NS | VF: 7 (27%)  VA: 4 (16%) | VF: 5 (71%)  VA: - | VF: -  VA: - | VF: -  VA: - | VF:  VA: 4 (100%) |
| Chabot (2015)^22^ | 0.7 months (range: 0.03-66.7) | 0-6 weeks | VF: 29 (74%)  VA: 23 (59%) | VF: 20 (69%)  VA:15 (65%) | VF: -  VA: - | VF: 0 (0%)  VA: 0 (0%) | VF: -  VA: - |
| Juraschka (2014)^36^ | NS | NS | VF: 37 (59%)  VA: 53 (84%) | VF: 34 (87%)  VA: 46 (73%) | VF: -  VA: - | VF: 0  VA: 3 (5%) | VF: 3  VA: 4 |
| Karppinen (2015)^37^ | NS | 3-6 months | VF: 33  VA: 35 | VF: 30 (94%)  VA: 31 (88%) | VF: -  VA: | VF: -  VA: 1 | VF: -  VA: - |
| Yildrim (2016)^46^ | NS | NS | VF: 32 (20%)  VA: 33 (20%) | VF: 14 (43%)  VA: 9 (27%) | VF: 14 (43%)  VA: 9 (27%) | VF: -  VA: - | VF: -  VA: - |
| **Studies reporting visual acuity (VA)** | | | | | | | |
| Constantino (2016)^25^ | NS | NS | VA: 12 (43%) | VA: 9 (75%) | VA: - | VA: - | VA: 2 (17%) |
| De Witte (2011)^6^ | NS | NS | VA: 37 (44%) | VA: 35 (95%) | VA: - | VA: 0 | VA: 2 (5%) |
| Fan (2014)^30^ | NS | NS | VA: 12 (43%) | VA: 12 (100%) | VA: - | VA: - | VA: - |
| **Studies reporting visual field (VF)** | | | | | | | |
| Anik (2011)^19^ | Full recovery: 14.7 (SD: 10.5), Partial: 50.1 (SD: 29.1), No recovery: 92.4 (SD: 15.4) | Second day and within 6 months | VF: 72 (100%) | VF: 58 (81%) | VF: 18 (24%) | VF: 0 (0%) | VF: 14 (19%) |
| Akin (2016)^10^ | NS | NS | VF: 52 (84%) | VF: 39 (75%) | VF: - | VF: 6 (12%) | VF: 5 (10%) |
| Cappabianca (1999)^21^ | NS | NS | VF: 4 (40%) | VF: 4 (100%) | VF: - | VF: - | VF: - |
| Chi (2013)^8^ | NS | NS | VF: 57 (71%) | VF: 47 (82%) | VF: - | VF: 0 (0%) | VF: 10 (18%) |
| Cho (2002)^23^ | NS | NS | VF: 8 (36%) | VF: 5 (63%) | VF: - | VF: - | VF: - |
| Dallapiazza (2015)^28^ | NS | NS | VF: 42 (53%) | VF: 31 (74%) | VF: - | VF: 0 (0%) | VF: 10 (24%) |
| Dehdashti (2008)^29^ | NS | NS | VF: 80 (40%) | VF: 71 (89%) | VF: 40 (50%) | VF: 0 (0%) | VF: 9 (11%) |
| D’Haens (2009)^27^ | NS | NS | VF: 4 (7%) | VF: 4 (100%) | VF: 2 (50%) | VF: - | VF: - |
| Leach (2010)^9^ | NS | 4-6 weeks | VF: 61 (69%) | VF: 54 (89%) | VF: - | VF: 2 (3%) | VF: 5 (8%) |
| Minet (2008)^41^ | NS | NS | VF: 31 (100%) | VF: 11 (35%) | VF: - | VF: - | VF: - |
| Nakao (2011)^42^ | NS | NS | VF: 43 (100%) | VF: 42 (98%) | VF: 17 (40%) | VF: - | VF: - |
| Paluzzi (2014)^11^ | NS | Immediately postoperatively and every 3-6 months for up to 2 years | VF: 237 (43%) | VF: 190 (80%) | VF: - | VF: 6 (3%)  VA: - | VF: 41 (17%) |
| Sheehan (1999)^44^ | NS | NS | VF: 12 (46%) | VF: 11 (92%) | VF: 2 (17%) | VF: 0 (0%) | VF: 1 (8%) |
| **Studies reporting unspecified general vision (GV)** | | | | | | | |
| Chohan (2016)^24^ | NS | NS | GV: 52 (84%) | GV: 39 (75%) | GV: - | GV: 6 (12%) | GV: 5 (10%) |
| Cusimano (2012)^26^ | NS | NS | GV: 26 (90%) | GV: 25 (96%) | GV: - | GV: - | GV: - |
| Gondim (2014)^32^ | NS | Immediately post-operatively, at 3, 6, 12 months and afterwards yearly. | GV: 24 (17%) | GV: 24 (100%) | GV: 14 (58%) | GV: 0 (0%) | GV: 0 (0%) |
| Gondim (2015)^33^ | NS | NS | GV: 48 (96%) | GV: 38 (79%) | GV: - | GV: 1 (2%) | GV: 9 (19%) |
| Ferreli (2014)^31^ | NS | NS | GV: 38 (69%) | GV: 33 (87%) | GV: - | GV: 0 (0%) | GV: 5 (13%) |
| Han (2013)^34^ | NS | 1 day, 3 and 6 months and annually afterwards | GV: 113 (45%) | GV: 102 (90%) | GV: - | GV: 0 (0%) | GV: 11 (10%) |
| Jho (1997)^35^ | NS | NS | GV: 3 (20%) | GV: 3 (100%) | GV: - | GV: 0 (0%) | GV: 0 (0%) |
| Koutourousiou (2013)^38^ | NS | NS | GV: 45 (83%) | GV: 36 (80%) | GV: 9 (20%) | GV: 2 (4%) | GV: 7 (16%) |
| Kuo (2016)^39^ | NS | NS | GV: 38 (100%) | GV: 27(71%) | GV: - | GV: 1 (3%) | GV: 10 (26%) |
| Marenco (2011)^40^ | NS | NS | GV: 24 (96%) | GV: 17 (71%) | GV: - | GV: 0 (0%) | GV: 7 (29%) |
| Sabry (2015)^43^ | NS | 1-3 months | GV: 21 (53%) | GV: 21 (100%) | GV: 17 (81%) | GV: 0 (0%) | GV: 4 (19%) |
| Wongsirisuwan (2014)^45^ | NS | NS | GV: 300 (79%) | GV: 27 (90%) | GV: - | GV: - | GV: - |
| Zhan (2015)^47^ | NS | NS | GV: 313 (100%) | GV: 250 (80%) | GV: 99 (32%) | GV: 5 (2%) | GV: 58 (19%) |

Supplementary table 3 legend: NS: not specified, VA: visual acuity, VF: visual field, GV: general vision
